# The role of clear lens extraction in angle closure glaucoma

**Published:** 2017

**Authors:** Vasile Potop, Catalina Corbu

**Affiliations:** * Clinical Ophthalmology Emergency Hospital Bucharest, Romania

**Keywords:** angle closure glaucoma, phacoemulsification, occult phacomorphic glaucoma

## Abstract

Clear lens extraction can be considered a therapeutic option in angle closure glaucoma (ACG). Even if it does not represent the first choice of treatment, it can be taken into consideration when the topical treatment does not control the intraocular pressure (IOP) and iridotomy does not have a positive effect on the angle closure, especially in appositional angle closure when biometry or ultrabiomicroscopy (UBM) show lens involvement.

In angle closure glaucoma, clear lens extraction represents an etiological treatment that takes into account the role of the lens in the pathogenesis of the disease. If we ignore it and we choose a filtrating surgery as therapeutic option we can end up with complications such as prolonged athalamia, corneal damage and lens opacification that will eventually require cataract surgery, but performed late and with higher risks. Before performing a filtrating surgery in ACG, we should take an UBM. We also need to choose the best moment to perform surgery, after topical treatment and iridotomy have been tested, but before trabecular damage appears.

## Introduction

In 2010 there were approximately 15,7 million people with angle closure glaucoma and about 4 million were at risk of total blindness [**1**]. Acute angle closure represents a form of disease, but the most frequent form is chronic angle closure. Even if its mechanism is already known, the disease can have a negative outcome [**2**]. These patients usually present with a more advanced neuropathy and the glaucoma progresses more rapidly than primary open angle glaucoma [**3**-**6**]. 

## Clinical aspects of angle closure

The risk of angle closure rises with the degree of angle closure because angles under 20 degrees may have parts with iridotrabecular contact. The degree of iridotrabecular contact represents criteria for prophylactic treatment in eyes with this conformation. Angle closure can be sectorial, complete, or intermittent. If there are more than 90 degrees of iridotrabecular contact, it is recommended to perform prophylactic treatment [**7**]. 

## Angle closure etiology

Among ACG, there can be primary angle closure glaucomas, with or without pupillary block, or secondary angle closure glaucomas. In primary angle closure glaucomas, angle configuration represents a common element. Associated with this configuration, we can find shallow anterior chamber, thick ciliary body or iris and small sagittal eye diameter [**8**,**9**]. For secondary angle closure, we mention iris traction and pupillary block.

## The role of the lens in primary angle closure

Angle closure is usually found in eyes with a special conformation: anterior chamber approximately 1 mm shallower than normal, narrow angle and lower sagittal diameter. Studies have shown an important lens involvement in these eyes [**10**,**11**]. The mechanism of angle closure can be an increased lens volume or anterior placement of the lens. Some authors reported that anterior lens placement represents about 60% of these cases and increased lens volume about 60% [**10**]. Also, it has been stated that lens vault might have a higher role in angle closure than an increased sagittal diameter of the lens or anterior placement of the lens. A continuous growth in lens volume and lens vault are suspected to be involved in the pathogenesis of angle closure especially in women in the 3rd or 4th decade [**12**]. 

Studies on patients with acute angle closure have shown in the other eye: shallow anterior chamber increased sagittal diameter of the lens, or angle closure. In 50% of the cases, these factors were considered decisive [**13**].

## The effect of lens extraction in ACG

Lens extraction has clear effects on the IOP and anterior chamber morphology. There are studies that show a decrease in the IOP with approximately 2mmHg, even in normal eyes, after phacoemulsification [**14**].

In primary open angle glaucoma, the IOP reduction after cataract surgery is higher in eyes with a higher preoperative IOP. In ACG, lens extraction can open the angle, deepen the anterior chamber, and decrease the iridotrabecular contact. These features have been associated with a stabile decrease in the IOP up to 12mmHg [**15**-**17**]. Some studies have also showed a decrease in peripheral anterior synechiae after lens extraction [**18**]. Preoperative factors with the highest predictive values were: initial IOP value and increased sagittal diameter of the lens [**19**,**20**]. 

## The effect of lens extraction in acute primary angle closure

Lam and col. conducted a study that compared the effects of peripheral laser iridotomy and early lens extraction in acute primary angle closure. The results showed that the IOP was lower and remained more constant in the lens extraction group than in the peripheral laser iridotomy group [**15**].

Another study showed that 2 years after acute primary angle closure, the IOP was lower than 22mmHg in 61,1% of the patients treated with topical and systemic medications and 98,5% of the patients who were submitted to phacoemulsification [**21**]. 

According to the American Academy of Ophthalmology, in cases of primary angle closure glaucoma, when the IOP is controlled with more than one topical medication, phacoemulsification can reduce the IOP in about 30% of the cases [**28**].

## The role of lens extraction in decompensated ACG

Many studies have shown that in patients in whom medication had little or no effect and associated cataract, combined cataract-glaucoma surgery had a higher effect on IOP reduction than lens extraction. Tham and col. revealed that in 3 months after surgery, the mean IOP was 14mmHg in the first group and 17mmHg in the second group, while at 18 months, the mean IOP was 13,2 mmHg in the first group and 15,4mmHg in the second group [**18**, **21**-**23**].

## Clear lens extraction in chronic angle closure

In a recent study, Tham and col. compared the effects of trabeculectomy with Mitomycin C vs. lens extraction in patients with chronic angle closure. They revealed that even if the results were similar in the two groups (an IOP reduction with 8,4 mmHg in 43% of the patients after trabeculectomy and 8,9mmHg in 36% of the patients after phacoemulsification), complications were higher in the trabeculectomy with Mitomycin C group. Also, about 1/ 3 of the patients who were submitted to trabeculectomy developed lens opacification [**24**]. 

## ACG refractory to conventional treatment

We can encounter some cases of primary angle closure glaucoma with an unfavorable outcome regardless of the treatment. This usually appears in relatively young females who present with high IOP values that do not respond to laser iridotomy or medical treatment. If we try to perform filtrating surgery, we might end up with complications such as prolonged athalamia or choroidal detachment. 

In these patients, UBM can reveal an increased sagittal diameter of the lens, which might explain why conventional treatment was not successful. Pupillary blockage in these patients appears trough lens involvement. The lens occupies almost the entire posterior chamber and it pushes the iris forward, this is why, in these cases, iridotomy has little effect, without plateau iris configuration. 

Transparent lens with normal volume, shallow anterior chamber, and a small eye diameter in an angle closure glaucoma patient, refractory to conventional treatment, should make us consider performing an UBM in order to evaluate the lens peculiarities. Small diameter of the eye represents the predisposing factor, while the lens is the determination factor. Of course, not all cases of angle closure glaucoma can be included in this group, but when we encounter a patient with these characteristics, who is unresponsive to medical treatment or laser iridotomy, we should consider this as occult phacomorphic glaucoma. 

In these patients, lens extraction seems to be the treatment of choice, but we need to take into consideration that the procedure is performed in a relatively young patient with transparent lens. Also, we need to carefully choose the moment of surgery, because, if we have a patient who used medical treatment for a long time and laser iridotomy had no effect, we might perform phacoemulsification with good anatomical results and still end up with high IOP due to peripheral anterior synechiae or trabecular damage. 

We cannot perform phacoemulsification as a routine treatment in angle closure glaucoma but we have to consider it in certain cases before irreversible damage appears. A proper IOP control, without or with minimal treatment, is to be expected if the surgery has been performed in the right moment.

## When is the right time to operate?

In cases in which UBM and anterior segment OCT have proven lens involvement, and medical treatment and laser iridotomy have little or no effect on the IOP, we should consider lens surgery. Studies have shown that peripheral laser iridotomy can stabilize the IOP in about 70% of the cases and medical treatment in about 84-99% of the patients. Also, prophylactic peripheral laser iridotomy was effective in almost 100% of the cases [**25**,**26**]. In Asian population, 6 months after peripheral laser iridotomy, 76,6% of the patients had high IOP values and 58,2% needed topical or surgical treatment to control the IOP [**27**].

In cases in which the IOP cannot be stabilized with laser iridotomy, the mechanism for angle closure might be plateau iris or it might involve some lens peculiarities. This is the right moment to further investigate the role of the lens in angle closure. Biometry can reveal an eye with a diameter less than, or about 22 mm with small anterior segment, or a nanophthalmic eye. These cases usually present as acute angle glaucoma or chronic glaucoma, with high IOP values uncontrolled with medical treatment or laser iridotomy. 

If laser iridotomy can control the IOP, it is not recommended to perform lens extraction, even if the lens is proven as an etiologic factor in angle closure.

## Conclusions

When the lens is proven as an etiological or a precipitating factor to a patient with primary angle closure glaucoma, who had little or no response to medical treatment and laser iridotomy, we need to consider phacoemulsification.

We also need to choose the best moment to perform surgery, after topical treatment and iridotomy have been tested, but before trabecular damage appears.

It is not indicated to perform clear lens extraction in every case of primary angle closure, or to use clear lens extraction as a first choice treatment. 
